# Crimean–Congo hemorrhagic fever epidemic during COVID‐19 in Iraq: A “double whammy”?

**DOI:** 10.1002/ccr3.6450

**Published:** 2022-10-17

**Authors:** Omer A. Shaikh, Manahil Shekha, Gulrukh Shaikh, Irfan Ullah, Zoia E. Khattak, Abdulqadir J. Nashwan

**Affiliations:** ^1^ Department of Medicine Ziauddin Medical University Karachi Pakistan; ^2^ Department of Medicine Liaquat National Hospital and Medical College Karachi Pakistan; ^3^ Department of Internal Medicine, Kabir Medical College Gandhara University Peshawar Pakistan; ^4^ Institute of Public Health and Social Science (IPH&SS) Khyber Medical University Peshawar Pakistan; ^5^ Khyber Medical College Peshawar Pakistan; ^6^ Hamad Medical Corporation Doha Qatar

Dear Editor,

*Hyalomma marginatum* ticks and farm animals transmit the Orthonairovirus that causes the Crimean–Congo Hemorrhagic Fever (CCHF). Due to the rising incidence of Crimean–Congo hemorrhagic fever across the globe, which has been long recognized in a vast geographic area, including Europe, Asia, and Africa, as well as the potential for viral growth into other regions, it has been a serious public health issue.^1^

As the world endeavors to overcome the devastating effects of COVID‐19 pandemic, which has already worn down the underfunded healthcare systems in the developing world, especially in countries such as Iraq, with more than 2.4 million confirmed cases and 25,000 fatalities as of September 21, 2022.^2^

Iraq has been notified of a Crimean–Congo hemorrhagic fever outbreak by WHO, which might prove to be a catastrophic calamity, particularly around the time of the year when Eid‐ul‐Adha draws near, and the majority of Iraq's health initiatives are focused on COVID‐19 control.^3^ By August 7, 2022, the country reported 1085 suspected cases of Crimean–Congo hemorrhagic fever since the year began. Out of the 1085 cases suspected, 287 were confirmed by polymerase chain reaction (PCR), 52 of which were fatal. Twelve of Iraq's 18 governorates indicated Crimean–Congo hemorrhagic fever prevalence, with the majority of cases occurring in Thiqar (42.5%) and Missan (11.1%), followed by Babil (8.4%) and Wasit (8.4%).^4^

Every year, during the Islamic occasion of Eid‐Ul‐Adha, countless livestock animals are slaughtered, including goats, cows, sheep, and camels. Worldwide, it is customary to buy animals ahead of Eid‐Ul‐Adha, most of which are kept in residential zones. In addition, untrained butchers, animal slaughter in public locations, improper disposal techniques, and handling of animal blood, tissues, and skin are some variables that aid in the transfer of Crimean–Congo hemorrhagic fever virus (CCHFV) from animal to human and pose a significant threat to the general populace.^5^

There is an urgent need for local authorities to implement formal regulation and sanctions to stem the flow of people and animals leaving regions where Crimean–Congo hemorrhagic fever is endemic. The health of animals in transportation should be checked and recorded, therefore veterinary authorities should establish checkpoints, control documentation, and immediately educate medical staff. Acaricide use and a 14‐day quarantine period prior to slaughter have proven effective in preventing tick infestation before animals are slaughtered, and people living in endemic areas should take extra care when handling animals and should always wear gloves and other personal protective equipment (PPE).

In addition, conducting public awareness campaigns and ensuring that local authorities effectively collect and dispose of waste and the carcasses of the slaughtered animals hold strategic importance in order to prevent the disease from becoming more and more burdensome during the current COVID‐19 pandemic, along with collaborative efforts between infectious disease researchers, medical professionals, and veterinarians for prompt diagnosis and management of Crimean–Congo hemorrhagic fever, are some of the crucial steps that could be taken at the earliest.

## AUTHOR CONTRIBUTIONS

Omer A. Shaikh, Manahil Shekha, Gulrukh Shaikh, Irfan Ullah, Zoia E. Khattak, Abdulqadir J. Nashwan involved in manuscript writing and editing. All authors read and approved the final manuscript.

## CONFLICT OF INTEREST

The authors have no conflicts of interest to declare.

## TRANSPARENCY DECLARATION

The lead author (Dr. Omer A. Shaikh) affirms that this manuscript is an honest, accurate, and transparent account of the study being reported; that no important aspects of the study have been omitted; and that any discrepancies from the study as planned (and, if relevant, registered) have been explained.

## Data Availability

Not applicable.
